# Fast *operando* X-ray pair distribution function using the DRIX electrochemical cell

**DOI:** 10.1107/S160057752000747X

**Published:** 2020-07-10

**Authors:** Maria Diaz-Lopez, Geoffrey L. Cutts, Phoebe K. Allan, Dean S. Keeble, Allan Ross, Valerie Pralong, Georg Spiekermann, Philip A. Chater

**Affiliations:** a Diamond Light Source Ltd, Diamond House, Harwell Science and Innovation Campus, Didcot OX11 0DE, United Kingdom; b ISIS Facility, STFC Rutherford Appleton Laboratory, Didcot OX11 0QX, United Kingdom; c School of Chemistry, University of Birmingham, Edgbaston, Birmingham B15 2TT, United Kingdom; d Narmandie Université, Ensicaen, Unicaen, CRNS, Crismat, Caen 14000, France; e Universität Potsdam, Institut für Geowissenschaften, Postdam 14476, Germany; f The Faraday Institution, Harwell Campus, Didcot OX11 0RA, United Kingdom

**Keywords:** *in situ* X-ray electrochemical cells, pair distribution function, total scattering, X-ray Raman spectroscopy

## Abstract

*In situ* electrochemical cycling combined with total scattering measurements can provide valuable structural information on crystalline, semi-crystalline and amorphous phases present during (dis)charging of batteries. *In situ* measurements are particularly challenging for total scattering experiments due to the requirement for low, constant and reproducible backgrounds. Poor cell design can introduce artefacts into the total scattering data or cause inhomogeneous electrochemical cycling, leading to poor data quality or misleading results. This work presents a new cell design optimized to provide good electrochemical performance while performing bulk multi-scale characterizations based on total scattering and pair distribution function methods, and with potential for techniques such as X-ray Raman spectroscopy. As an example, the structural changes of a nanostructured high-capacity cathode with a disordered rock-salt structure and composition Li_4_Mn_2_O_5_ are demonstrated. The results show that there is no contribution to the recorded signal from other cell components, and a very low and consistent contribution from the cell background.

## Abstract 2

The Diamond Radial In Situ X-ray (DRIX) cell for combining electrochemical cycling with total scattering measurements is presented and applied to X-ray pair distribution function and X-ray Raman scattering studies on the high-capacity cathode material Li_4_Mn_2_O_5_.

## 1. Introduction   

The global increasing demand for sustainable energy delivers a formidable challenge to develop new electrode materials for rechargeable batteries with ever-increasing energy densities, longer cycle durability and minimal environmental impact, which operate safely and at low cost. State-of-the-art electrode materials suffer from capacity fading, chemical reactions at the particle surface, and structural degradation with detrimental effects on the battery performance and lifespan. These limitations have motivated the use of a wide variety of techniques to understand structure–property relationships and enable the design of new electrodes with improved performance. Such studies are challenged by the complexity of batteries. The active materials in the battery (*i.e.* anode, cathode – often themselves complex multi-component materials – and electrolyte) are assembled into a cell stack, which is also in contact with other parts of the cell (current collectors, separators, *etc.*). During (dis)charging of the cell, charged cations are transferred between the electrode materials, whose structures accommodate a large number of defects coupled to changes in the oxidation state of transition metals or anions which can also lead to phase separation or amorphization (Grey & Tarascon, 2016 ▸).

Common *ex situ* battery studies consist of the offline electrosynthesis of several samples cycled to different states of charge. However, not only is this approach resource- and time-consuming, but the disassembly of batteries for *ex situ* sample recovery can often result in phase contamination (*e.g.* oxidation) even when prepared in inert glovebox atmospheres, and the relaxation of highly reactive intermediates leading to misleading interpretation of what is actually happening during electrochemical cycling. This problem can be avoided using *in situ* and *operando* studies, which probe the structure of electrode materials during the electrochemical reaction. Although technically more complex, *in situ* studies allow reliable identification of short-lived and highly reactive intermediates, as well as increasing the reliability of the data by continuously monitoring the evolution of a single sample rather than multiple samples individually prepared. Therefore, where possible, battery materials should be studied under realistic conditions.

*In situ* battery studies at synchrotron sources are becoming more routine using a range of techniques. For instance, X-ray absorption spectroscopy (XAS) including near-edge (XANES) and extended (EXAFS) regions have been applied to study the change of oxidation state and local structure of selected atoms in materials (McBreen, 2009 ▸). X-ray photoelectron spectroscopy (XPS) has allowed chemical states at battery interfaces to be probed during cycling (Wu *et al.*, 2018 ▸). Bragg diffraction has offered insights into the structural changes of crystalline phases (Johnsen & Norby, 2013 ▸; Peterson *et al.*, 2017 ▸), and when used in combination with computed tomography methods can allow 3D crystallographic mapping within a battery (Finegan *et al.*, 2020 ▸). Imaging methods allow for the direct visualization of battery components in terms of microstructure, morphology and chemical composition (Wolf *et al.*, 2017 ▸; Ulvestad *et al.*, 2014 ▸). The speed of modern synchrotron imaging even allows data to be collected during catastrophic cell failures, and combination with suitable *in situ* apparatus allows concurrent collection of calorimetric data and ejected media from explosive events (Finegan *et al.*, 2019 ▸).

Each technique provides information on different physical/chemical aspects of batteries over different length-scales and is used in combination to reach a full understanding of these complex multi-component systems. However, battery materials can undergo complex phase transformations upon cycling which may involve the formation of intermediate phases with low crystallinity (Stratford *et al.*, 2017 ▸) that may go undetected by the above techniques. Although sensitive to amorphous phases, the interpretation of interatomic distances from EXAFS is model dependent because of the scattering phase-shift and only provides information on the nearest atomic shells (≤4–5 Å). Total-scattering methods (combining Bragg and diffuse scattering) allow recovery of information about the local structure and amorphous phases embedded in the diffuse scattering data, which are disregarded in conventional Bragg diffraction. This technique probes the bulk average information of true interatomic distances and is sensitive to all length scales from local-, meso- to long-range ordering.

Total scattering and pair distribution function (PDF) techniques have been pivotal in observing important changes in the structure of a range of battery materials. The sensitivity of the PDF method to atomic short-range ordering has been key to identifying the effect that cation ordering can have on the conductivity of cathodes with spinel (Liu *et al.*, 2016 ▸) or rock-salt structures (Jones *et al.*, 2019 ▸). Complex phase transformations involving amorphous intermediate phases have also been successfully investigated: up to five intermediate phases of tin anodes could be identified by PDF (Stratford *et al.*, 2017 ▸), and the *in situ* formation of an Fe^II^_(1−*x*)_Fe*_x_* ^III^O*_x_*F_(2−*x*)_ nanocomposite could be detected (Wiaderek *et al.*, 2013 ▸). In addition, PDF is highly complementary to – and is often studied in combination with – traditional crystallographic studies of average structures, and local probes with atomic selectivity such as XAS and nuclear magnetic resonance (NMR) spectroscopy (Zeng *et al.*, 2007 ▸; Bréger *et al.*, 2005 ▸).

Despite the unique information afforded by such experiments, *in situ* PDF studies are less common than those employing *in situ* Bragg diffraction or XAS. This is not only due to the later development or accessibility of the technique, but more notably the experimental constraints associated with *in situ* PDF studies. High-quality PDF data require the data to be collected over a wide range of momentum transfer (*Q*), necessitating the combination of high X-ray energy and a detector with large angular coverage. The scattered X-ray signal at high *Q* is very weak because of the fall-off of the X-ray form factor with *Q*; therefore, the combination of high flux and highly efficient detectors (covering a large area and consisting of high-*Z* materials) are needed. Background contributions from air scattering and any containers must be subtracted to obtain scattering from just the sample; the background signals should be minimized and above all be repeatable to facilitate the subtraction. Although some laboratory measurements are possible using Ag and Mo source instruments, the best-quality X-ray PDF data are collected at synchrotron sources where high energy and flux are available. There are a number of specialized PDF beamlines with sufficient flux for time-resolved experiments, including, but not limited to, 11-ID-B at the Advanced Photon Source, 28-ID-1 at NSLS-II, P02.1 and P07 at PETRA III, ID11 and ID15A at ESRF, and I15-1 XPDF at Diamond Light Source, which is the focus of this study.

Ideally, *in situ* PDF experiments of batteries should be carried out on cells with low scattering intensity relative to the sample, which allows the measurement of data over a wide angular range in order to access the high *Q* necessary for good quality PDF data. PDF data collection requires the acquisition of the experimental background with a very high accuracy in order to successfully remove the contributions from the air scattering, sample container, sample holder, Compton scattering, *etc.*, all of which are highly challenging when the sample is buried in a complex sample environment like a battery. However, the majority of *in situ* PDF studies reported were performed in cells that were specially designed for Bragg diffraction or XAS where experimental constraints are less demanding; Bragg diffraction only considers sharp reflections while fitting a background empirically, and XAS is element-specific and therefore tolerant of background signal.

Standard cells for electrochemical testing, like coin and pouch cells, can be easily adapted for *in situ* experiments via simple modifications in their design to incorporate X-ray transparent windows (Richard, 1997 ▸). Thin polyimide (*e.g.* Kapton) or polyester (*e.g.* Mylar) tapes are frequently used for harder and softer X-rays, respectively. However, when using flexible and non-conductive windows, such a simple modification could disrupt the electrochemical reactions of the material in contact with the window, as demonstrated in previous work (Borkiewicz *et al.*, 2015 ▸). This could produce misleading results, as the small region of electrode probed does not match the performance of the ensemble of the battery. The non-conductive character can reduce the electrical contact of the cell with the electrode, and the flexibility of the tape can result in uneven pressures applied to the battery stack and deformation, which will in turn complicate the acquisition of consistent backgrounds for PDF (Borkiewicz *et al.*, 2015 ▸). Furthermore, Kapton windows rarely provide sufficiently good hermetic protection, leading to potential for leaking of the electrolyte and contamination of the cell by air.

The combination of computed tomography with PDF (CT-PDF) (Jacques *et al.*, 2013 ▸) offers great advantages for removing the scattering contribution from the battery cell. However, CT-PDF studies of batteries are time intensive even at modern synchrotron sources; they therefore lack the fast time resolution of the *operando* X-ray PDF approach introduced in this paper, and remain rare (Sottmann *et al.*, 2017 ▸).

Kapton windows have been replaced by rigid and conductive low-*Z* elements, like glassy carbon or beryllium, in other cell designs (Leriche *et al.*, 2010 ▸; Sottmann *et al.*, 2016 ▸; Borkiewicz *et al.*, 2012 ▸; Hartung *et al.*, 2015 ▸), offering an improved seal, stack pressure and conductivity for *in situ* XAS and Bragg diffraction experiments. These multipurpose electrochemical cells, AMPIX (Borkiewicz *et al.*, 2012 ▸) or Bruker (Leriche *et al.*, 2010 ▸), are also based on more standard coin and Swagelok cells, respectively. In these configurations, the active material is pressed against the window resulting in a small sample thickness. In axial-geometry cells, the incoming X-ray beam is perpendicular to the cell stacking direction and therefore sees all the layers in the cell including the positive and negative electrodes, the separator, the electrolyte, and even other parts of the cells (outgoing window, springs, *etc.*). Thus, the signal from the thin layer of material of interest is buried in the measured data. In order to increase the signal from the layer of interest, the layer thickness may be increased; however, this can pose a challenge to the electrochemical performance of the battery as the achievable cycling rate of the battery is often decreased and the larger thickness may make structural and chemical inhomogeneity within the layer more significant. Although it is possible to carry out *in situ* PDF in such cells (Hua *et al.*, 2014 ▸, 2013 ▸), data processing is far from facile and is restricted to strongly scattering samples for which the signal can be approximately deconvoluted from the measured data. For example, a structural evolution study of amorphous carbon electrodes on such cells is highly challenging, where, to make matters worse, the windows are made of the same material. Moreover, these cell configurations are also sensitive to other changes in the cell outside the material of interest (*e.g.* varying composition and thickness of electrode layers or metal foils, parasitic electrolyte reactions, electrochemical reactions of the window material, *etc.*) and it is difficult to correct for a changing background during PDF experiments. Reliable acquisition of background is hindered by low reproducibility of the positions of the battery layers and X-ray attenuation by the sample.

This work introduces the newly designed Diamond Radial In Situ X-ray (DRIX) cell recently commissioned at the I15-1 XPDF beamline at Diamond Light Source, which builds on previous work on radial-geometry cells (Stratford, *et al.*, 2017 ▸; Liu *et al.*, 2016 ▸, 2020 ▸; Young *et al.*, 2017 ▸). The total scattering signal from the component of interest (*i.e.* cathode, anode or solid electrolyte) can be isolated by focusing the X-ray beam on that layer of the cell. This radial geometry allows for battery layers to be kept thin and for optimized electrochemical performance while the signal from the layer is determined by the radius of the cell; the result can be an order of magnitude increase in the volume of the layer of interest probed in radial geometry when compared with typical axial-geometry cells. The new DRIX cell consists of thin-walled fused quartz tubes and low-*Z* rods acting as current collectors. Such design minimizes the parasitic scattering and absorption from the cell, and allows the collection of high-quality total scattering and PDF data, even on weakly scattering or amorphous phases, in only a few minutes.

Using the nanostructured Li_4_Mn_2_O_5_ disordered rock-salt cathode as an example, we illustrate the improvement of signal purity versus other radial-geometry cells. We demonstrate the ability to efficiently probe multiple battery cells during a single experiment with excellent data quality comparable with *ex situ* data acquisitons.

## 2. Cell design   

**Figure 1:**
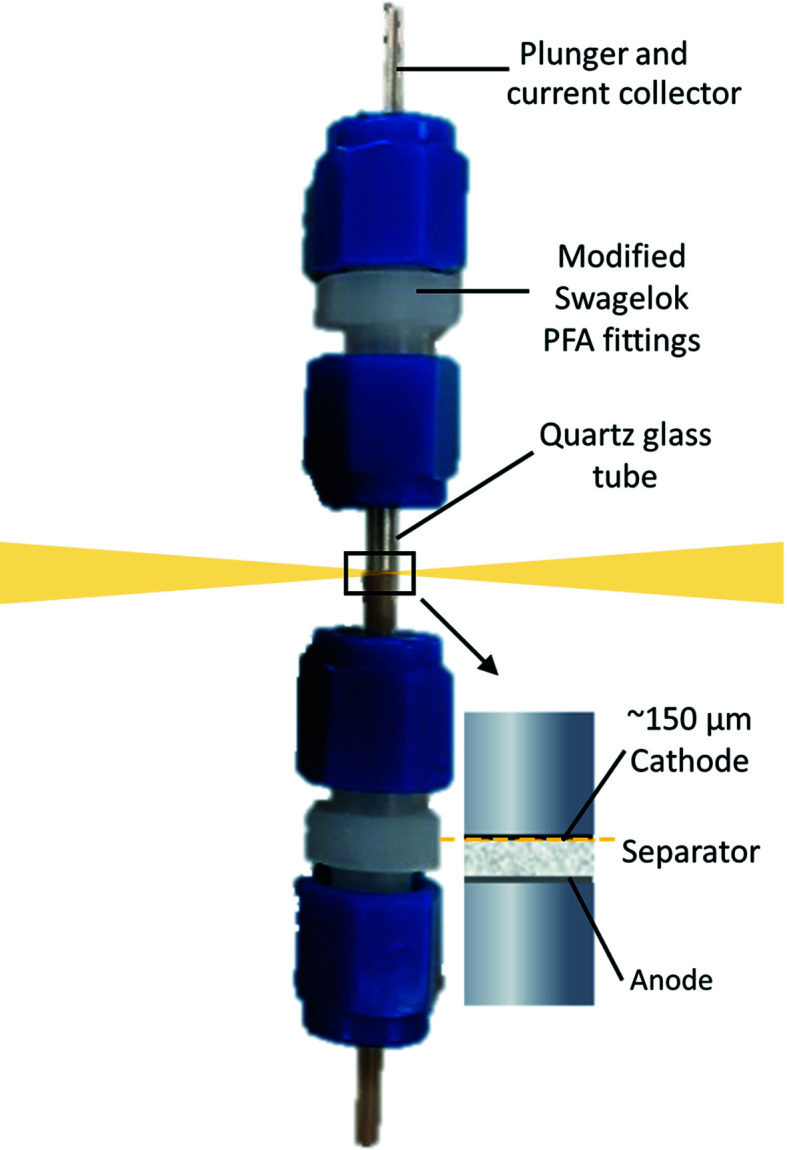
Photograph of an assembled cell with a schematic representation of the electrode stack impregnated in electrolyte (inset). The incoming and transmitted X-ray beams are represented in yellow.

Radial-geometry cells are the preferred option for straightforward background corrections of *in situ* PDF studies of batteries, and can be used to study a single layer component, or can be used for depth profiling along the cell-stacking direction. Given how in battery systems the anode, electrolyte and cathode are often complex multi-component systems, and how PDF data are one-dimensional bulk probes of all the materials present in the X-ray beam, the ability to isolate some parts of the battery is especially useful. In this design, the layers of the battery stack are oriented parallel to the X-ray beam as shown in Fig. 1 ▸. As a result, the incoming X-rays travel along the battery stacked layers increasing the active material thickness probed from ∼0.2 mm typical in axial-geometry cells to ∼3 mm for the DRIX radial-geometry cells.

### 2.1. Battery body   

Previous radial cells have used X-ray transparent tubes as the battery body to provide wide angular access for X-rays. The choice of material could be insulating like Kapton (Liu *et al.*, 2016 ▸) or perfluoro­alk­oxy (PFA) (Stratford *et al.*, 2017 ▸), or a conducting carbon fibre capillary (Young *et al.*, 2017 ▸) also acting as the working electrode. The more widespread choice of Kapton bodies or windows for *in situ* cell designs has disadvantages associated with long-term exposure to electrolyte and mechanical stability. PFA can also suffer from mechanical instability and deformation; it is hard to machine so is generally relatively thick (up to 1 mm) and adds significant features in the Bragg data which can be difficult to accurately subtract from the background.

The battery body of the DRIX cell is composed of a thin-walled (<100 µm) fused-quartz glass tube with a 2.9 mm inner diameter and a Swagelok union at each end of the tube which provide a hermetic seal to the cell. The use of an insulating material greatly simplifies the cell design (Young *et al.*, 2017 ▸) and improves the overall air-tightness with respect to Kapton (Liu *et al.*, 2016 ▸). In addition, the optical transparency of the fused-quartz glass helps to visualize the battery stack for an easy assembly and alignment with the X-ray beam.

**Figure 2:**
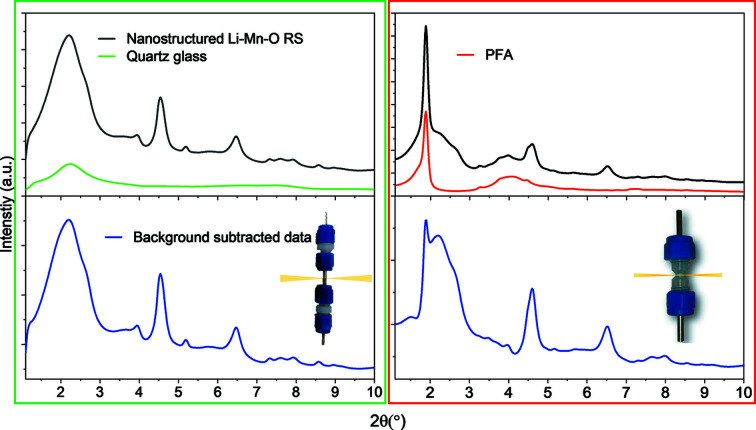
*In situ* XRD patterns for nanostructured LiMnO:C composites in a 7:3 ratio showing no signal (either Bragg or diffuse) from other cell components (*i.e.* current collectors, fibre glass separators or lithium metal). The background measured for an empty cell is overlapped and the background-subtracted signal is given below. It should be noted that the intensities of the datasets are normalized to an absolute scale. Thus, the artefacts in the PFA background-subtracted data originated from the technical challenges of manufacturing PFA cells with homogenously thin walls, where the scattering from PFA cells varies as a function of the cell orientation making the background data collection more challenging.

Fig. 2 ▸ shows *in situ* data collections and backgrounds for the DRIX cell and compares it with a previous version using PFA as the battery body. The absence of sharp Bragg reflections in the diffraction pattern of the glass yields significant advantages for background subtraction and PDF data processing. Moreover, fused-quartz glass is a non-flexible material and the capillaries used are manufactured with a high reproducibility, aiding background repeatability across different cells, a further advantage over Kapton and PFA RATIX cells (Liu *et al.*, 2016 ▸; Stratford *et al.*, 2017 ▸).

### 2.2. Electrodes   

The typical cell assembly includes a regular electrode stack consisting of a negative electrode, an electrolyte-soaked glass fibre separator and a positive electrode. The components of the electrode stack are prepared with a smaller diameter of ∼2.85 mm than typical coin or Swagelok cells. The electrode can be either freestanding, pelletized or deposited on metal foil.

### 2.3. Current collectors   

**Figure 3:**
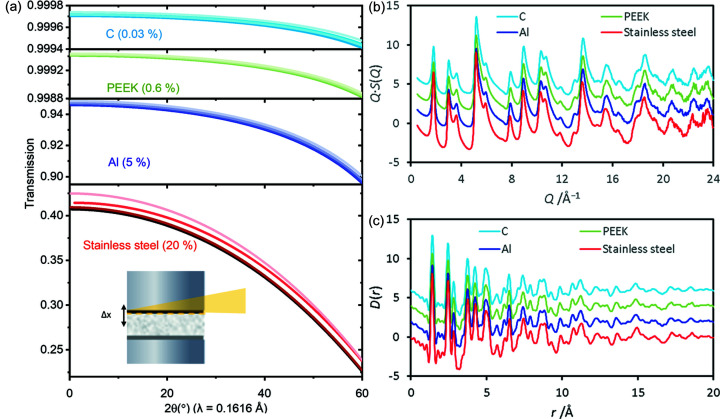
Minimization of the parasitic scattering from the current collectors. (*a*) X-ray transmission for carbon, PEEK, Al and stainless steel current collectors as a function of the scattering angle. The percentage change between low and high angle is given in the graphs. The X-ray transmission is given as a function of the distance of the beam from the current collector (Δ*x*, as shown in the inset) when the beam is at the edge of the current collector: 100 µm, 500 µm and 1 mm away from it (from dark to light shades, respectively). (*b*) Total scattering data, *Q* × *S*(*Q*); and (*c*) PDF data for glassy carbon collected with carbon (blue), PEEK (green), Al (purple) and stainless steel (red) current collectors.

During a measurement, the DRIX cells are aligned to record the signal from the electrode within the vicinity of the current collectors. The proximity of the beam with typical current collectors made of strongly scattering metals (*e.g.* steel, copper or aluminium) gives rise to the sloped background shown in Fig. 3 ▸, which is difficult to reproduce experimentally during cell background acquisitons. Therefore, although the currect collectors do not see the direct beam, we have improved their design to futher decrease the absorption effects from these cell components resulting in improved accuracy of the scattering data and simplified data corrections.

Fig. 3 ▸(*a*) shows the absorption profile for 76.69 keV X-rays (the most common working energy of the I15-1 XPDF beamline) as a function of the scattering angle for current collectors made from stainless steel, and some lower-*Z* materials: aluminium (Al), polyether ether ketone (PEEK), and glassy carbon (C). Stainless steel provides good chemical compatibility with most electrodes and electrolytes, but has a strong absorption profile and is therefore not optimal. Al displays significantly less absorption, but is not compatible with all cathode materials. PEEK provides good chemical compatibility, but is not conducting; PEEK rods with an ∼3 µm metal (copper or aluminium) coating prepared via virtual cathode deposition (Plasma App Ltd, UK) provide good conductivity for operation as a current collector. Vitreous (glassy) carbon rods (Hochtemperatur Werkstoffe GmbH, Germany) provide the lowest absorption profile and show good conductivity, but are also prone to intercalation of Li^+^ ions. It is possible to add a thin layer of aluminium foil between the current collectors and the active electrode material to aid cell preparation and alignment. The absorption when using the lower-*Z* materials is considerably less, allowing more accurate background subtraction with no further corrections. Total scattering and PDF data from amorphous carbon (Sigma–Aldrich, 99.95%) collected inside a DRIX cell with stainless steel, Al, Cu-coated PEEK and vitreous C current collectors are shown in Figs. 3 ▸(*b*) and 3(*c*), respectively. Data in the range 0.5 Å^−1^ ≤ *Q* ≤ 24 Å^−1^ were corrected and processed to PDFs using the program *GudrunX* (Soper, 2011 ▸). The total scattering data collected with a stainless steel current collector [Fig. 3 ▸(*b*)] were hard to normalize during PDF processing and showed appreciably more noise at *Q* > 20 Å^−1^, owing to higher attenuation through the steel at high scattering angles. The higher absorption from steel also causes a shift in the PDF data to higher *r*, which could lead to misleading results. This shift is because absorption from the steel results in less scattering observed from the edge of the sample, where the X-rays first enter the sample, and more from the exit edge, resulting in a shift in the centre of mass of sample scattering closer to the detector and an apparent larger lattice parameter. Although in theory these absorption effects could be deconvoluted from the data, it is better to avoid them completely and use a current collector made of a low-*Z* material.

## 3. Implementation on I15-1 XPDF   

### 3.1. Total scattering measurements   

The data reported in this paper were acquired at the I15-1 XPDF beamline at Diamond Light Source (UK) using 76.69 keV radiation. A 2D Perkin Elmer XRD4343CT detector was positioned 20 cm away from the multi-cell holder to maximize the *Q*-range for optimal PDF data quality. A dark (without X-rays) detector image was collected at the start of the experiment to determine the dark current contribution to the data, which was subtracted from all subsequent data; the detector was kept in a constant read-out state and air-cooled with fans to maintain a constant temperature, which led to negligible changes to the dark current contribution during the experiment. The collected diffraction patterns were background corrected using diffraction patterns of empty cells and carbon (present in the active cathode mixture to improve electron conducting properties), where the carbon background was also collected in a cell for maximum precision of the corrections.

DRIX cells were aligned by scanning the cell in the vertical direction to a position close to the current collector. The cell positions were fine-tuned to optimize the scattering from the active material but at a distance where the signal from the current collector is no longer observed. For this, the beam height is focused to a value below the thickness of the active material (FWHM ≃ 8 µm), and it is carefully aligned to probe the region below the interface between the current collector and active material so that only the active material contributes to the measured data, as depicted in the inset of Fig. 1 ▸. Given the size of the beam is ∼8 µm in the vertical direction and ∼700 µm in the horizontal direction, this cell orientation allows for the detection of X-ray scattering from a single battery layer component and depth profiling of the cell stack.

### 3.2. Electrochemical setup for up to ten cells   

The electrochemical (eChem) setup on the I15-1 XPDF beamline uses a potentiostat (Ivium n-STAT) which allows the study of up to eight *in situ* measurements concurrently. This system has been integrated into the beamline control software *EPICS* (Mooney *et al.*, 2000 ▸) and the data acquisition software *GDA* (OpenGDA, 2020 ▸). The eChem system allows for multiple samples (*e.g.* doping levels) or experimental timescales (*e.g.* charging rates) to run concurrently, greatly increasing the efficiency of beam time and the breadth of the studies that can be performed on the beamline.

**Figure 4:**
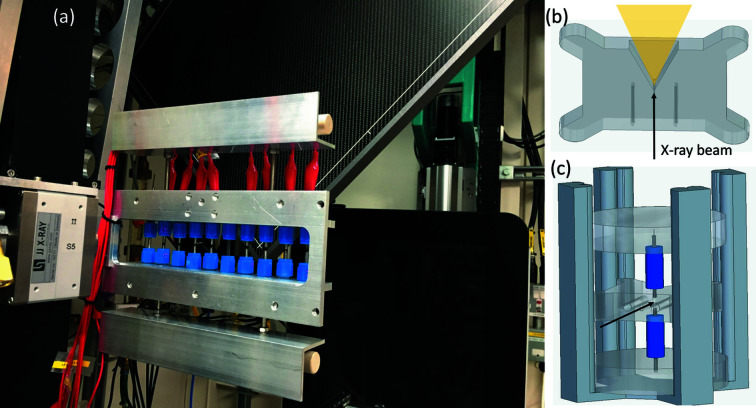
(*a*) *In situ* eChem battery setup at the I15-1 XPDF beamline. (*b*) Single DRIX cell heater; the incoming beam is represented by an arrow and the transmitted X-ray beam is represented in yellow. (*c*) Assembled DRIX cell using the heater and a specially designed frame for easy alignment.

The multi-stage electrochemical setup in Fig. 4 ▸(*a*) consists of ten evenly spaced stages holding the DRIX cells in the vertical direction. The multi-stage holder consists of a stainless steel frame where the batteries are attached and fixed in place by the tightening of fixing screws holding onto the Swagelok fittings. Thus, the cells can be easily dismounted and swapped for others during an experiment. DRIX cells are connected to the potentiostat using crocodile clips that are attached to the plungers acting as current collectors. Cables are managed by two L-shaped shelves in the top and bottom parts of the frame that keep the cables out of the beam-path. Proper cable handling not only avoids the unplugging of cables during moves to swap between different battery positions but can also prevent the shadowing of the beam by loose cables which would hinder the processing of PDF data.

During an experiment, X-ray scans can be alternated between the different cells by an easy displacement of the multi-stage holder along the directions normal to the X-ray beam. Since the frame is assembled in one piece, the shift between different batteries along the direction of the beam is negligible, which simplifies the alignment of cells. During an experiment, up to eight of the ten batteries in the multi-stage holder are connected to the potentiostat and cycled. The two remaining positions are typically used to calibrate the holder position with a silicon standard and to measure an empty cell for background subtraction. However, given the reproducibility of the sample stage position after an initial calibration with a standard and background collections, up to ten batteries could be mounted in the stage ready for cycling.

### 3.3. Cell heating   

Cell heating improves both ion mobility and solid-state diffusion, and is particularly important in systems with slow reaction kinetics. A custom designed heater in Fig. 4 ▸(*b*) allows DRIX cells to operate at a wide temperature range from room temperature up to 180°C. The possibility to customize the operating temperature during the experiments allows the study of liquid electrolyte cells at ≤55°C (below boiling or evaporation of the liquid electrolyte), and at ≤170°C for all solid-state batteries (below the melting of lithium metal at 180°C).

## 4. Cell performance   

The electrochemical performance of the DRIX cell has been benchmarked against standard coin and Swagelok cells using identical battery preparation procedures and cycling conditions. The tests were performed using freestanding powder in the absence of binders or additives that could extend the battery lifetime. The cells have been successfully run for up to 70 h (Diaz-Lopez *et al.*, 2020*a* ▸) (the longest period of time they were tested for to date), showing a high cycling stability comparable with other *in situ* cells (Borkiewicz *et al.*, 2012 ▸). Extended cycling of batteries is crucial to aid the proper understanding of these systems to inform the development of new, long-lasting battery materials.

## 5. Experimental   

### 5.1. Materials   

Nanostructured cation-disordered Li_4_Mn_2_O_5_ rock-salts were synthesized by a two-step route using mechano­chemical activation. First, high-temperature LiMnO_2_ was synthesized by a solid-state reaction using a reagent mixture of LiOH (≥98%), MnO_2_ (99%) and MnO (99%) with the molar ratio of 2:1:1, and was heat-treated at 1000°C under an argon flow for 8 h. Then, the mechanochemical synthesis between LiMnO_2_ and Li_2_O in a 2:1 ratio was carried out using Fritsch Planetary Micro Mill PULVERISETTE 7 premium at 700 rpm for 20 h to produce nanostructured Li_4_Mn_2_O_5_.

### 5.2. Electrochemical measurement   

The electrochemical measurements were performed using lithium as a counter electrode. The composite electrodes were prepared by grinding the rock-salt nanopowders with carbon black (weight ratio of 7:3). A 1 *M* LiPF_6_ solution in EC (ethyl­ene carbonate) and EMC (ethyl methyl carbonate) with a volume ratio of 3:7 was used as the electrolyte. The cells were assembled in an argon-filled dry glove box with a typical loading of 1–2 mg of the active material. The electrochemical studies were carried out at room temperature using an Ivium-n-STAT potentiostat at a slow cycling rate of extraction/insertion of C/40 (obtaining a theoretical capacity of 350 mAh g^−1^ in 40 h) operating in galvanostatic mode between 2.0 V and 4.5 V. This slow rate, close to thermodynamic conditions, was used to aid the understanding of electrochemical processes involved during (dis)charge cycles.

## 6. Results   

### 6.1. Total scattering   

Most readily available *in situ* cells are not optimized for PDF experiments, limiting the systems investigated to those with sufficient scattering to obtain useable PDF data. We demonstrate here the quality of the X-ray scattering signal from the DRIX cells suitable for PDF analysis of even amorphous or poorly scattering samples. The setup was used to study a nanostructured high-capacity cathode displaying a disordered rock-salt structure with the composition Li_4_Mn_2_O_5_ (Freire *et al.*, 2016 ▸). The investigation of the structural evolution of this system is highly challenging due to the nanostructuring by mechanochemical alloying and the high concentration of defects. The structure of pristine Li_4_Mn_2_O_5_ can be seen as an Mn–O type rock-salt where 2/3 of the Mn cations are replaced by Li leading to the formation of 1/6 O vacancies (□) that are surrounded by Li in a highly distorted □Li_6_ octahedral configuration (Diaz-Lopez *et al.*, 2018 ▸). Upon charging, 75% of Li (50% of the cations) are removed from the material without unfavourable phase transformations.

**Figure 5:**
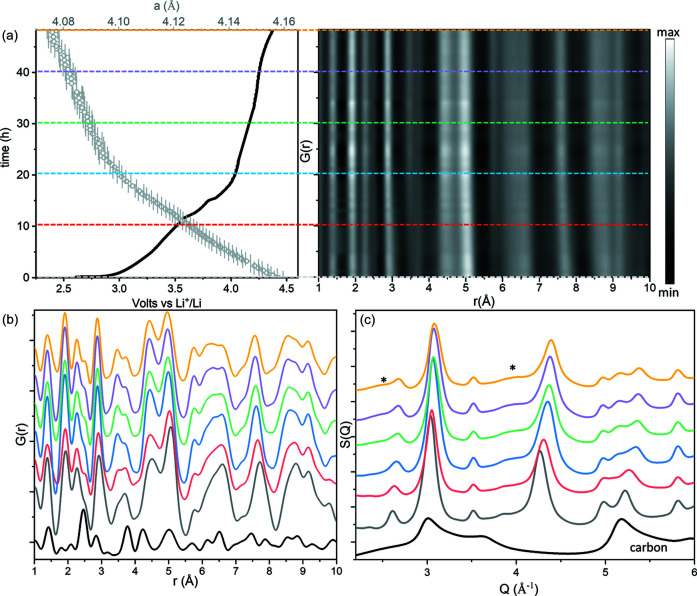
*Operando* total scattering data of Li_4_Mn_2_O_5_:C composites. (*a*) Electrochemical performance (solid line) overlaid with refined lattice parameters (empty circles). PDF data were recorded during cycling. (*b*) PDF and (*c*) *S*(*Q*) functions at selected times [indicated by dashed lines in (*a*)] overlaid with the signal from carbon. The asterisks highlight diffuse scattering attributed to the *in situ* formation of a short-range-ordered superstructure.

Fig. 5 ▸ shows the full (local and average) structural evolution of Li_4_Mn_2_O_5_ over the course of the first charge to 4.4 V. The electrochemical performance recorded in the DRIX cells displays a solid solution behaviour with the absence of plateaus characteristic of phase transformations, which is confirmed by the absence of secondary phases (amorphous or crystalline) in the total scattering data. The lattice parameter evolution of the rock-salt phase quantified by the Rietveld refinement of an average structural model fitted simultaneously to the long *r*-range of the PDF (15–30 Å) and reciprocal space data showed a continuous evolution that mirrors the shape of the electrochemical curve. The refined lattice parameter of the rock-salt phase contracts from 4.158 (2) Å in the pristine phase to 4.070 (2) Å in the charged material, in agreement with previous reports (Freire *et al.*, 2018 ▸). Data collected for only 2 min were background-subtracted and corrected to calculate the PDFs in Fig. 5 ▸, which are also in agreement with previous *ex situ* data from the work by Freire *et al.* (2018 ▸).

As previously discussed, only the capillary and the battery layer under investigation are illuminated by the primary beam, so no contributions from other parts of the cell (lithium metal, separator or current collectors) are observed in the scattering data. Since only the signal of an empty capillary was considered as the sample background, the steps and datasets necessary to calculate PDFs measured in *operando* conditions using the DRIX cell on the I15-1 XPDF beamline are identical to *ex situ* data collections in conventional capillaries. It is worth noting that even, if the capillaries in the cell are larger than the typical capillaries used at the beamline (2.95 mm versus 1.0–1.5 mm), the *Q*-resolution of the cell datasets is still acceptable. The *Q*-resolution can be further increased by the recording of data with a second area detector available on the I15-1 XPDF beamline and located further away from the sample (∼90 cm). The distance between the sample and detector can be optimized for different systems in order to find the best compromise between the *Q*-range and *Q*-resolution.

The evolution of the coherent scattering signal *S*(*Q*) during cycling shows the formation of broad features in the data that can be ascribed to the ordering of Li/Mn cations. The evolution of these broad features in the data could be successfully identified owing to the accurate background subtraction allowed by the DRIX cells. The specific structural changes are discussed in detail elsewhere (Diaz-Lopez *et al.*, 2020*b* ▸).

### 6.2. X-ray spectroscopy   

The DRIX cell was originally optimized for X-ray PDF studies, but it could equally be used for *in situ* spectroscopic studies. *In situ* XAS performed on the *K*-absorption edge of transition metals is extensively used to study the electronic and local structure of electrode materials (McBreen & Balasubramanian, 2002 ▸). The penetration of hard X-rays, combined with some of the advantages of the technique (*e.g.* it is element specific, requires small sample volumes and short data collections) make *in situ* XAS battery experiments on *K*-edges of transition metals accessible. On the other hand, the *K*-edges of light elements such as lithium or oxygen, or the transition metal *L*/*M*-edges are found within the soft X-ray regime. *In situ*/*operando* soft X-ray spectroscopy in batteries is highly challenging due to the short penetration depth of soft X-rays which are only sensitive to the surface, and the requirement of a vacuum environment (Liu *et al.*, 2013 ▸) which hinders the bulk characterization in real battery environments. Alternatively, bulk characterizations of light elements could be performed by X-ray Raman scattering (XRS). In XRS, an X-ray photon transfers energy to a core electron, exciting it to an unoccupied state; excitation of core electrons requires energies of ∼10 keV, much higher than the corresponding *K*-edge spectroscopies on light atoms. The process is analogous to XAS, only instead of energy from an absorbed X-ray photon it is the energy transfer from the excitation which is studied; this makes the process analogous to Raman scattering. In this way, XRS combines the advantages of a hard X-ray probe (when compared with other spectroscopic methods) with the sensitivity of soft-XAS, enabling bulk studies under more challenging sample environments and in *operando* (Bergmann *et al.*, 2002 ▸; Braun *et al.*, 2015 ▸). So far, the XRS spectra of Li, C and O *K*-edges of different battery components (electrolyte solutions, anode and cathode materials) (Ketenoglu *et al.*, 2018 ▸; Gog *et al.*, 2009 ▸; Chan *et al.*, 2011 ▸) have been investigated *ex situ*. Obtaining XRS data with a good signal-to-noise requires access to a wide angular range from the sample. XRS is also a bulk probe, meaning that geometries where multiple battery components are illuminated by the incident X-ray beam may give rise to complex and overlapping XRS data, which cannot be analysed. These requirements make previous battery designs for XAS/diffraction (Leriche *et al.*, 2010 ▸; Sottmann *et al.*, 2016 ▸; Borkiewicz *et al.*, 2012 ▸) with low angular range and signal purity unsuitable.

**Figure 6:**
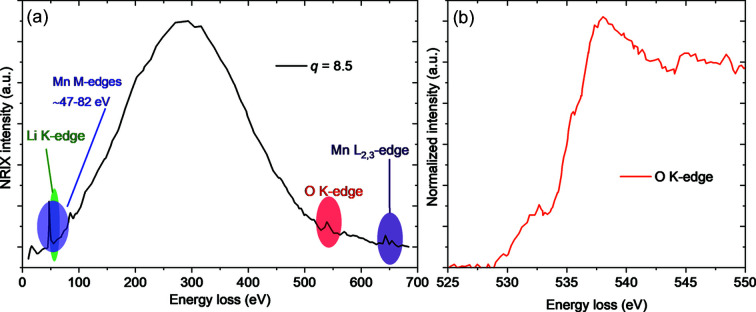
X-ray Raman scattering data. (*a*) Wide scan of Li_4_Mn_2_O_5_ at coarse steps showing the richness of the excitation spectra covering Mn (*M* and *L*_2,3_ edges) as well as Li and O *K*-edges. (*b*) Normalized O *K*-edge spectra.

The DRIX *in situ* cell introduced here can be readily utilized for XRS to propel this technique towards *operando* data collections, with the added advantage that in this cell configuration the sample thickness can be varied by the off-centering of the capillary to optimize the XRS signal (*ex situ* tests performed at ID20-ESRF, see Fig. 6 ▸). Note that in XRS, as in XPDF, the improvement of signal purity in radial-geometry cells versus transmission cells is important to spatially resolve the signal from an element (*e.g.* Li, O) simultaneously present in the electrolyte, cathode and anode layers. We expect to deconvolute the signal from the Li/O in the active material to that from the liquid electrolyte by measuring different cell components in isolation followed by background subtraction (analogous to the processing of PDF data) (Balasubramanian *et al.*, 2007 ▸). Bulk-sensitive XRS of light elements (Li, O, C: 1*s*) as well as transition metals (TM: 2*p*, 3*p* and 3*s*) could potentially be performed on the same cell with identical electrochemistry.

## 7. Conclusions   

The DRIX cell design represents a new oportunity to conduct high-quality structural studies of electrode materials using total scattering and PDF methods and will soon find applications in other spectroscopic methods such as XRS. These bulk multi-scale measurements will provide a deeper insight into the multiple processes happening within a cycling battery.

The low and constant background scattering from the DRIX cell provides excellent total scattering data quality, comparable with *ex situ* data collections. Application of these cells on the I15-1 XPDF beamline at Diamond Light Source, in combination with a multi-cell holder and multi-channel potentiostat, allows for concurrent cycling and sequential data collection of up to eight batteries. Cable management and beamline design means that the background from the holder is minimized. Using these new characterization tools it is possible to develop a comprehensive understanding of reaction processes within battery materials including knowledge of the short-range ordering, nanostructured materials and characterization of amorphous intermediate phases.
